# Advances in the study of the relationship between Alzheimer's disease and the gastrointestinal microbiome

**DOI:** 10.1002/ibra.12065

**Published:** 2022-09-08

**Authors:** Xin‐Yan Li, Hao‐Yue Qin, Ting‐Ting Li

**Affiliations:** ^1^ Southwest Medical University Luzhou Sichuan China; ^2^ Department of Anesthesiology, Institute of Neurological Disease, West China Hospital Sichuan University Chengdu China; ^3^ Department of Anestheiology, West China Tianfu Hospital Sichuan University Chengdu China

**Keywords:** Alzheimer's disease, gastrointestinal microbiome, gut hormones, probiotics, short‐chain fatty acids

## Abstract

There are many trillions of bacteria in the gastrointestinal microbiome (GM). Their ecological dysregulation can contribute to the development of certain neurodegenerative diseases, including Alzheimer's disease (AD). AD is common dementia and its incidence is increasing year by year. However, the relationship between GM and AD is unclear. Therefore, this review discusses the relationship between GM and AD, elaborates on the possible factors that can affect this relationship through the inflammation of the brain induced by blood−brain damage and accumulation of amyloid deposit, and proposes feasible ways to treat AD through GM‐related substances, such as probiotics, Mega‐3, and gut hormones, including their shortcomings as well.

## Graphical abstract

Alzheimer's disease (AD) is a common degenerative disease faced by humans and its prevalence has been increasing recently, but there is no exact cause and mechanism of its development. This review discusses how and why abnormalities in the gastrointestinal biome may contribute to AD and the treatments associated with it.

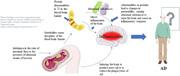

## 1 INTRODUCTION

Alzheimer's disease (AD) is a progressive neurodegenerative disorder with an inapparent onset and slow progression. Since AD currently accounts for 60%−80% of dementia cases, its rapid rise in prevalence is drawing attention to it.^1^ Clinically, it is distinguished by a broad range of dementia symptoms, including but not limited to memory impairment, aphasia, dysgraphia, executive dysfunction, and personality and behavioral changes. The pathophysiology of AD is yet unknown, though.

The gastrointestinal microbiome (GM) refers to the genetic composition of all microorganisms in the human gastrointestinal tract. According to incomplete statistics, GM includes 2000 different strains or species, the number of which is even close to 100 trillion. Although the majority of them are beneficial or neutral to the host, it cannot be excluded that some microorganisms are harmful to the host and that they may play some role in maintaining host health or certain diseases.^2^, ^3^, ^4^ Because these microorganisms contain more than 3 million genes, they can produce thousands of metabolites, which then regulate the body, such as exogenous and drug metabolism, maintenance of the structure of the intestinal mucosal barrier integrity, and immune regulation and resistance to pathogen invasion.^5^

The possibility that GM may contribute to the etiology of several neurodegenerative illnesses became increasingly apparent as research developed.^6^, ^7^, ^8^ Currently, the commonly held belief is that AD is associated with cognitive impairment and brain β‐amyloid (Aβ) accumulation. Recently, laboratories in Italy and the United States have indicated the discovery of an interaction between brain protein misfolding and the microbiome.^9^, ^10^, ^11^ It has also been demonstrated that the composition of GM in people of different ages is different. For instance, humans have certain microbial genomes within their GM that secrete specific glycans just during infancy.^12^ In comparison with the young people, the number of thick‐walled tissue and bacillus‐like bacteria is increased in the elderly. These findings point to some co‐evolution of hosts and microbes.^13^ Besides, it has been proved that there are experiments showing that altered gut microbial diversity does affect the development of AD.^14^, ^15^ We speculate that changes in GM in the elderly are responsible for the development of AD.

From the current studies, there are several hypotheses about the possible effects of GM on AD that this review summarizes based on previous research and will argue around the following three points: (1) GM may affect the integrity of the blood−brain barrier (BBB); (2) GM may cause brain injury or brain inflammation, leading to brain damage, which may then be converted to AD; and (3) GM may affect the accumulation of amyloid in the brain. And we also further explore the corresponding therapeutic measures and application prospects from the mechanism.

## 2 EFFECTS OF THE GM ON THE BRAIN

According to recent studies, it has been found that microbiota seems to directly affect the central nervous system (CNS) through the brain−gut axis (BGA). The BGA connects the CNS to the external environment and contains the GM as its main component. Its primary function is to regulate the entry of food and microbial metabolites into the organism's intestinal barrier, as well as the sympathetic and parasympathetic arms of the autonomic nervous system, the enteric nervous system, and the vagus nerve, which transmits signals to the brain.^16^, ^17^, ^18^ Because there is a bidirectional connection between the brain and the gut, the brain can influence the activity in the gut through the nervous system, and microbial secretions in the gut can bind to the nerves through the axis.^19^ The vagus nerve connects the nerves of the gut to the nerves of the brain, and since more than 90% of the vagal fibers are input, the possibility of influencing the CNS through the gut is high. According to the current research progress, the gut ecological disorder may cause neurocognitive disorders, such as depression, anxiety disorder obsessive‐compulsive disorder, and dementia, and it can affect the memory and learning ability of the brain as well as hippocampal plasticity.^20^ Therefore, it is possible that the gastrointestinal microbiota affects the brain leading to the development of AD.

### 2.1 Impairment of the BBB leads to the development of AD

**Figure 1:**
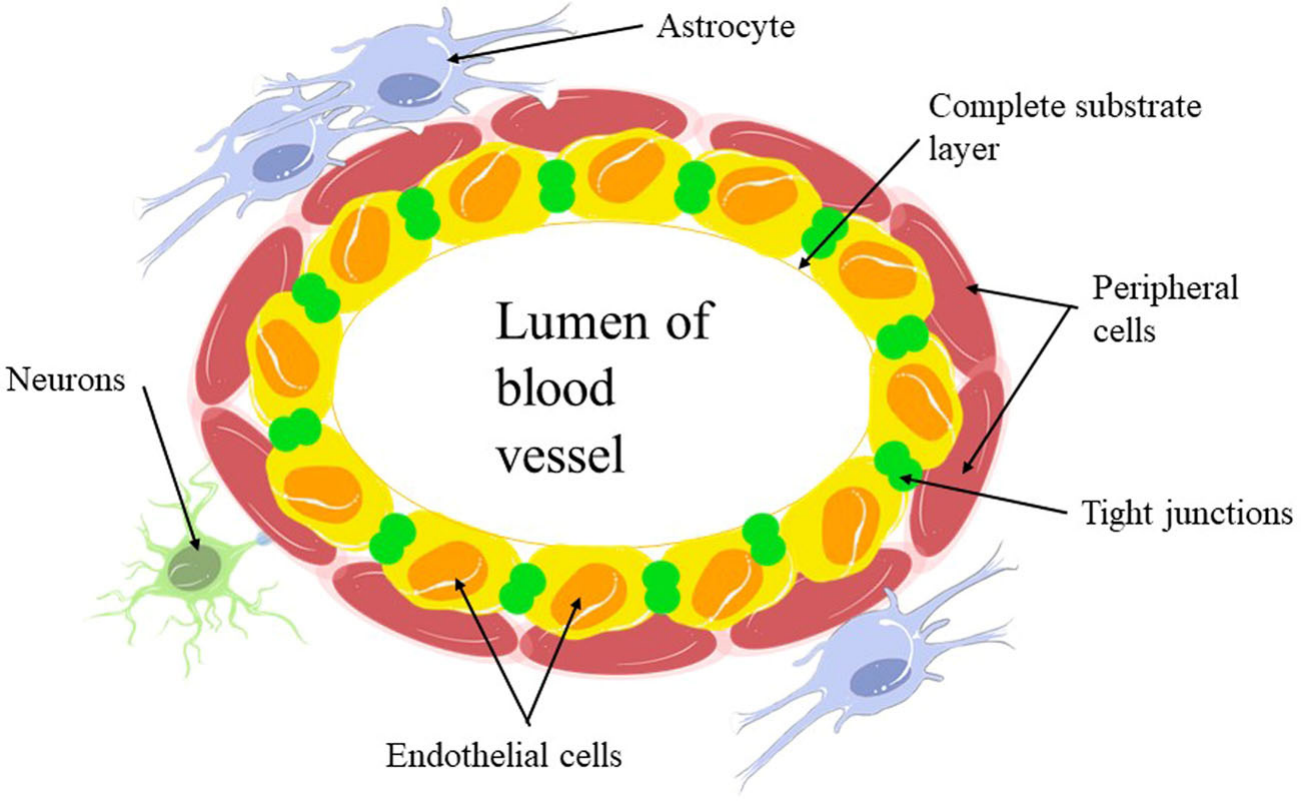
Structure of the blood−brain barrier [Color figure can be viewed at wileyonlinelibrary.com]

As shown in the structure of the BBB, it can be seen that the endothelial cells are connected by tight junctions (TJs), surrounded by pericytes, and surrounded by the basal lamina in its entirety (Figure 1).

There are TJs between endothelial cells attached to the intact basal lamina and surrounded by pericytes. The phenomenon of Aβ deposition in the cerebrovascular system is defined as cerebral amyloid angiopathy (CAA).^21^ CAA has been shown to lead to the degeneration of pericytes and endothelial cells,^22^ while brain microvascular endothelial cells (BMECs) can regulate the physical and functional integrity of the BBB.^23^ Combined with the schematic diagram of the BBB structure, we can speculate that the target of BMECs action may be present in one of the structures on the diagram, such as TJs or endothelial cells. Thus, we speculate that if CAA occurs in the brain, then BMECs may be impaired, leading to disruption of the integrity or certain functions of the BBB.

It has been suggested that TJ has a role in avoiding the random diffusion of proteins between membrane compartments while guiding cells from the blood to the CNS. Accordingly, if TJ is faulty, it may lead to the entry of chemicals, such as harmful compounds that enter the circulation due to intestinal damage, which may lead to severe brain damage. When disease occurs in the CNS, the expression of TJ proteins and the activity of the proteins themselves are affected. Occludin, a transmembrane protein found inside the TJ, acts primarily to anchor cytoplasmic band occludin (ZO) proteins 1 and 2 and the plasma membrane of neighboring cells.^24^ Claudin forms the backbone of the TJ, while occludin is in the claudin structure and has a function involved in regulating permeability.^24^ In impaired intestinal barrier function, Guo et al. found downregulation of ZO1 and occludin. Therefore, in CNS, if ZO1 and occludin appear downregulated it also indicates impaired BBB.^25^ Occludin is recognized as a sensitive indicator of structural changes in the BBB during disease pathogenesis.^26^ And also has been demonstrated to cause alterations in TJ proteins.^27^ Based on this, we propose that the changes in TJ protein caused by Aβ may lead to changes in the properties of transmembrane proteins like Occludin, resulting in increased permeability of the BBB.

A notable example of an error in transport due to an altered protein on the BBB is the glucose transporter‐1 (GLUT1). GLUT1 is lower in the brains of AD patients or mouse models than in the corresponding normal controls.^28^, ^29^ The loss of GLUT1 function will result in the incorrect transfer of glucose into the brain, leaving the brain tissue undernourished and potentially hypofunctional.^30^ Besides, Winkler et al.^31^ found that GLUT1 deficiency leads to BBB disintegration in mice. Precisely, its deficit resulted in a reduction in connexin in the TJ, directly affecting BBB permeability and significantly increasing the risk of brain injury.^31^, ^32^, ^33^ Although it is not clear why GLUT1 expression is reduced in AD patients, it is apparent that its reduction not only leads to an insufficient supply of nutrients in the brain but also to BBB disintegration when this reduction occurs in the endothelium.^33^, ^34^

A number of studies using mice as animal models have shown that damage to the GM in mice can impair the function of the BBB to some extent and even lead to the loss of certain functions, alter cortical myelin formation and hippocampal neurogenesis, lead to reduced cognitive function and affect memory formation, and reduce social behavior.^35^, ^36^, ^37^, ^38^ Since these occurrences are remarkably similar to the clinical signs of AD in humans, we believe that GM can induce AD by disrupting the BBB. The role of the BBB as a barrier between blood and cerebrospinal fluid (CSF) in systemic circulation (including the stomach) is crucial. It prevents most microorganisms and toxins from entering brain tissue from the blood and maintains the relative stability of the internal environment of the brain, thus protecting the normal function of the CNS and reducing the probability of brain injury. As a result, if the BBB is damaged, some toxic compounds created by GM can reach the brain. Therefore, the elevated BBB permeability may allow the harmful substances produced by GM to cause brain damage and lead to the development of AD.

### 2.2 Peripheral inflammation may damage the brain or cause inflammation in the brain leading to the development of AD

In the brains of two models of AD (5xFAD and 3xTg‐AD mice), large numbers of neutrophils have been observed to migrate into the brain and secrete the cytokine leukocyte factor 17 (IL‐17). In addition to secreting, IL‐17 releases neutrophil extracellular traps (NETs) and other inflammatory mediators.^39^ The involvement of these inflammatory mediators and NETs is not clear at present. However, it is evident that NETs are present in the brains of AD patients and, by depleting neutrophils, improve memory loss in AD mice.^39^ In addition, a 6‐month follow‐up of several older adults with AD who had acute systemic inflammatory events (SIEs) demonstrated an increase in cognitive decline due to acute systemic inflammation associated with elevated TNF‐α.^40^

Some studies have shown that it may be the case that after inflammation of the brain in AD patients, glial cells enter the area of the brain where inflammation exists thus clearing cells and plaques, but in fact, when these glial cells reach that area, they are forced to release more harmful Aβ and form more deposits, thus attracting more glial cells and entering such a vicious cycle.^41^ However, whether such a cycle is real remains unclear, but based on the results of Balin and Hudson,^42^ we can at least be sure that Aβ attacks the brain during inflammation caused by microbial infections.

The presence of chronic inflammation in the AD brain has been demonstrated, and it has also been documented that monocytes are infected with *Chlamydia pneumoniae* (Cpn) within the AD brain, so we assume that there is some possibility that the brain inflammation is caused by Cpn infection of monocytes during the pathogenesis of AD.^43^

**Figure 2:**
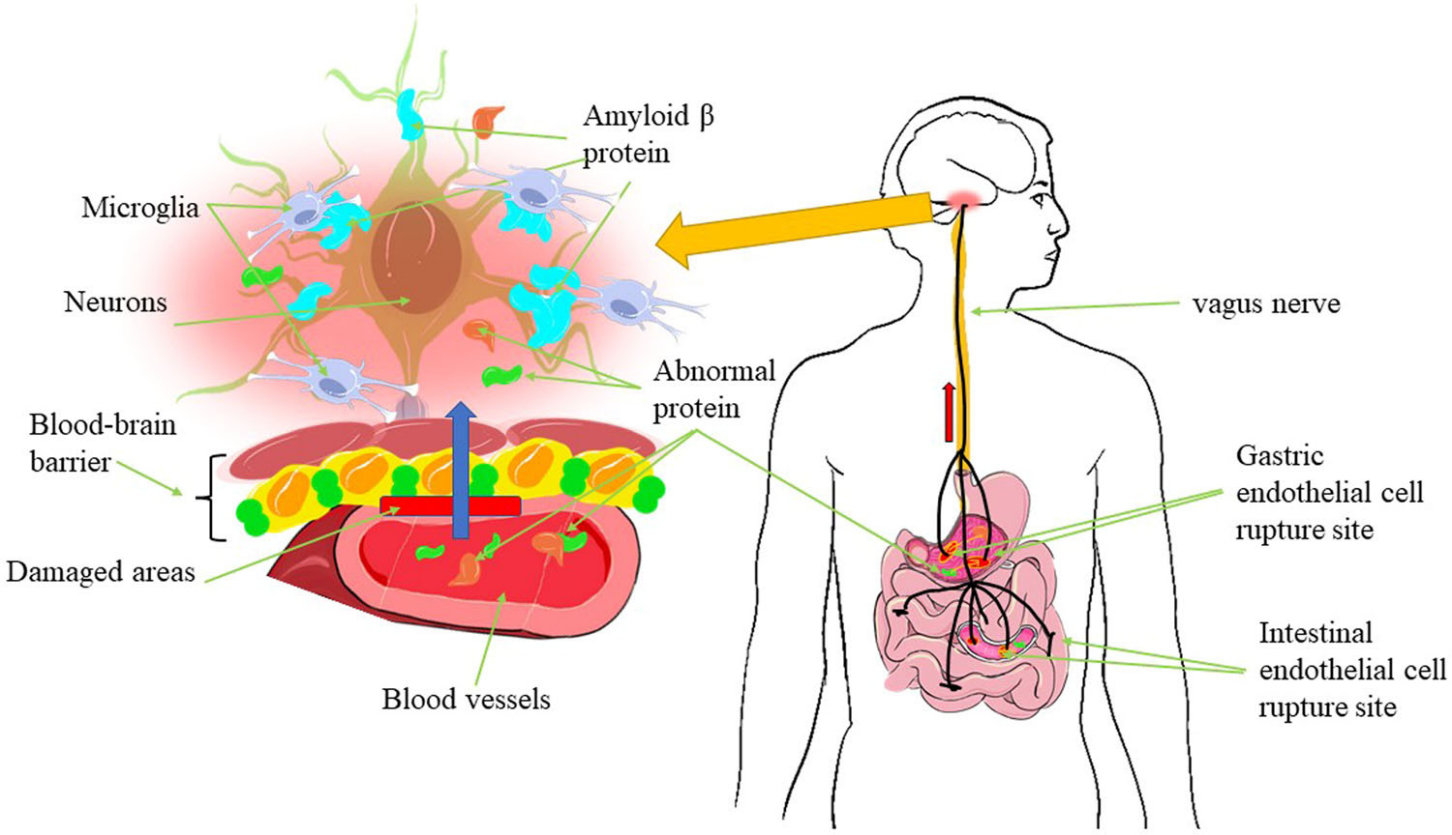
Schematic diagram of gut microbes triggering brain inflammation [Color figure can be viewed at wileyonlinelibrary.com]

Furthermore, by the schematic diagram of the gut microbiota and neuroinflammatory axis in AD proposed by Rashad Alkasir et al. (Figure 2), we suggest that when there is inflammation or a disruption of the barrier in the gut, some microbial abnormal metabolites may access the brain through the vagus nerve or other pathways, causing brain injury.

Some abnormal metabolites produced by microorganisms in the gastrointestinal tract may enter the circulation through the broken intestinal epithelium and enter the brain through the blood from the posterior cerebral region or through afferent nerve pathways (e.g., vagus nerve), thus causing neuroinflammation. It is also conceivable that certain aberrant compounds entering the brain may lead to changes in the permeability of the BBB, allowing more abnormal substances to enter the brain and causing more neuroinflammation.

### 2.3 GM affects brain amyloid deposition and induces AD

It has been shown that microorganisms from aged APPPS1 mice were colonized into the gastrointestinal tract of germ‐free APPPS1 mice, and a significant increase in Aβ levels in the brain of germ‐free APPPS1 mice was found, whereas the gut biome of aged wild‐type mice colonized in the same manner did not exert the same effect.^10^

In comparison with the conventional transgenic APPPS1 mice, whose brains already showed substantial brain Aβ deposition, the age‐matched germ‐free mice showed significantly less dense Aβ deposition in their brains, suggesting that no further Aβ deposition would occur without the influence of certain microorganisms.^10^ That is, the GM of mice with Aβ deposition do have pathogenic strains that promote Aβ deposition.

In an in vivo mouse model of Aβ amyloidosis, long‐term induction with an antibiotic mixture (ABX) alters the intestinal microbial diversity, and Aβ plaque deposition appears to be greatly decreased in male APP_SWE_/PS1_ΔE9_ transgenic mouse model.^9^ Chemokine 11 (CCL11) levels are elevated in the serum of ABX‐treated mouse models.^9^ We hypothesize that higher CCL11 levels may be caused by ABX reducing the population of certain microbes in the gut. Since CCL11 may pass across the BBB on its own, the concentration of CCL11 inside the BBB may also increase, activating the microglia and speeding up the phagocytosis of Aβ to minimize Aβ accumulation in the brain.^44^

Moreover, recent studies in AD transgenic mouse models have revealed that manipulation of the gut microbiota can affect brain amyloid deposition.^9^, ^10^ Therefore, we believe that if deposition of Aβ directly affects the brain leading to AD, GM can indirectly contribute to the development of AD.

## 3 POSSIBLE RELATIONSHIP BETWEEN GM AND THE OCCURRENCE OF AD

### 3.1 Microbial products may have some effect on AD

Ninety‐five percent of the human microbiome is likely to be found in the gastrointestinal flora. Approximately 70%−80% of human immunological tissue is contained in the intestinal mucosal lymphoid tissue, making it the biggest and most significant human immune organ.^45^

A large amount of lymphoid tissues around the mucosa is in close contact with the GM for a long time, and there are abundant bacteria in the GM. Bacteria secrete certain specific compounds or metabolites, so the microbiota in the GM may also secrete some neuroactive molecules, which may affect the brain.^46^ Bifidobacterium can influence the central transmission of 5‐hydroxytryptamine, a neurotransmitter that has been observed to promote autonomic function, by Candida, Streptococcus, *Escherichia coli*, and Enterococcus, and high levels of 5‐hydroxytryptamine can improve cognitive performance in the brain.^47^, ^48^ Lactobacillus and Bifidobacterium produce the inhibitory neurotransmitter γ‐aminobutyric acid, which acts at approximately 30% of the synaptic sites in the CNS, and some Lactobacillus produce acetylcholine, which specifically binds to certain receptors, thereby affecting membrane permeability and ion concentrations on both sides of the membrane.^49^ On the other hand, norepinephrine is an agonist of β‐adrenaline. It can alter the production of an LTP‐dependent protein that can regulate LTP and also affect the production of dopamine receptors, which coincidentally, are produced by bacteriophages. Also, it has been documented that both norepinephrine and dopamine are affected in AD.^50^, ^51^ Hence, we speculate whether the production of these products is responsible for the development of AD.

### 3.2 Tryptophan‐related metabolism may be associated with neuritis that induces AD

Tryptophan is generally considered to be an indispensable prerequisite for the coordination of gastrointestinal physiology and CNS function through the regulation of indole derivatives, the kynurenine pathway, and 5‐hydroxytryptamine synthesis.^48^, ^52^ The relationship between imbalances in the 5‐hydroxytryptamine and kynurenine pathways and a variety of neurodegenerative diseases as well as AD has been identified in several previous studies. Furthermore, dysregulation of the tryptophan pathway related to the kynurenine pathway is then considered to be a major contributor to AD, and indole‐3‐pyruvate, a metabolite of tryptophan, has been identified as a marker for identifying and predicting AD. On the other hand, we believe that the intestinal barrier and the BBB may be due to dysbiosis of the gastrointestinal flora, allowing metabolites of intestinal microorganisms that should not get to the brain to penetrate into the brain with the blood.^53^ These metabolites can directly or indirectly disrupt the BBB causing neuroinflammation, and prolonged neuritis may lead to neurodegeneration or brain damage, resulting in the development of AD.^54^, ^55^

### 3.3 Altered levels of short‐chain fatty acids (SCFAs) and bile acids may be associated with the development of AD

Metabolomics of fecal microbiota identified three classes of metabolites that are perturbed in AD patients, namely tryptophan, SCFAs, and bile acids.^56^ Additionally, seven SCFAs, including formic acid, acetic acid, propionic acid, butyric acid, 2‐methyl butyric acid, valeric acid, and isovaleric acid, showed a decreasing trend from the amnestic mild cognitive impairment (MCI) group to the AD group, with six of the seven SCFAs (formic acid, acetic acid, propionic acid, 2‐methyl butyric acid, isovaleric acid) were significantly lower in amnestic mild cognitive impairment (aMCI) and AD, whereas valeric acid was significantly lower in AD.^56^ Besides, they found that there are substantial variances in five SCFAs, including formic acid, acetic acid, propionic acid, 2‐methyl butyric acid, and isovaleric acid.^56^ In conclusion, their findings imply that alterations in fecal SCFAs are associated with disease progression in AD. As for lithocholic acid (LCA), an increase in fecal LCA was found in AD patients, as well as an increase in LCA in both patient serum and brain tissue of AD mice, indicating that intestinal‐derived LCA has an influence on AD pathogenesis.^56^

## 4 POSSIBLE WAYS TO TREAT OR ALLEVIATE AD THROUGH THE GM

### 4.1 Treatment of AD with probiotics

It has been found that certain probiotics can affect the BGA in certain ways, thus causing some effects on the CNS.^57^

Distrutti et al.^58^ found that VSL#3 attenuated the age‐related decline in LTP by performing LTP in a mouse model that had been administered VSL#3. Since LTP is thought to be associated with altered synaptic strength, changes in synaptic strength led to changes in memory. They therefore concluded that VSL#3 regulates the expression of a specific mediator that can alter synaptic plasticity in the hippocampal region of aged rats.^58^

**Figure 3:**
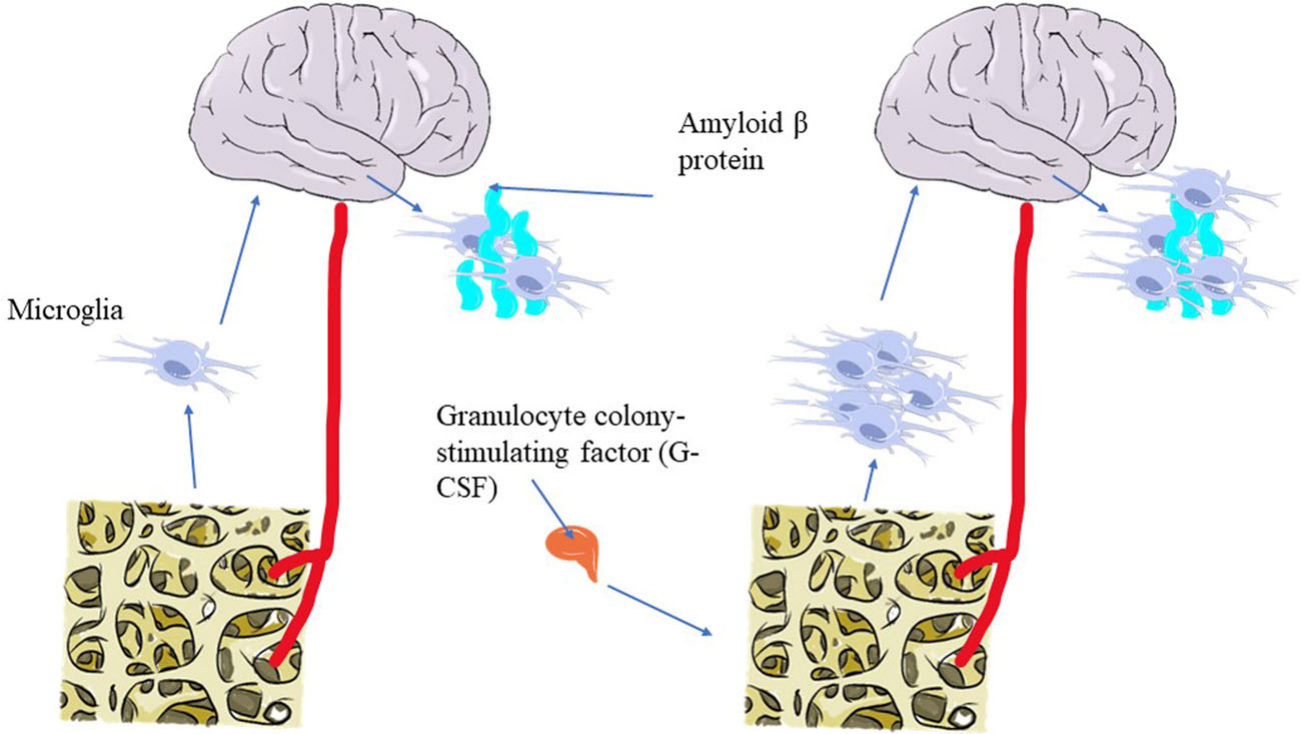
Schematic representation of the principle of granulocyte colony‐stimulating factor to reduce Aβ deposition in the brain [Color figure can be viewed at wileyonlinelibrary.com]

Kobayashi et al.^59^ investigated the effects of Bifidobacterium short strain A1 on behavioral and physiological processes in AD mice and found that Bifidobacterium short strain A1 reduced cognitive impairment in Aβ‐injected mice. Aβ injection is an injection into the ventricles of mice thereby causing symptoms such as a considerable decline in learning and memory capacity, and can establish a model that mimics the pathological features of AD in many ways. Since the model established by Aβ injection into mice is a more ideal animal model, for now, Bifidobacterium holds tremendous promise for therapeutic treatment of cognitive impairment in AD. Following genetic profiling of the hippocampus in mice, it was concluded that *Bifidobacterium breve* strain A1 may be involved in modulating the excessive immune response induced by Aβ injection to achieve improvement in Aβ toxicity. According to blood tests following treatment, *Bifidobacterium breve* strain A1 may also be improving cognitive levels by increasing plasma acetate. The change of microbiota in probiotic‐administered patients, on the other hand, has been proven to provide an anti‐inflammatory impact. Granulocyte colony‐stimulating factor (G‐CSF) was significantly higher in AD mice treated with this approach, and this factor has been shown to reduce and even reverse cognitive deficits in Aβ deposition in AD mouse models (Figure 3).^60^

G‐CSF may stimulate the bone marrow to produce more microglia, which can act as phagocytic agents for Aβ deposition. The more microglia, the less Aβ deposition.

Recently, it has been proved that when probiotic pretreatment was administered to mice in the presence of chronic psychological stress, changes in hippocampal neurogenesis guided by the water avoidance stress (WAS) in the mouse brain improved and the expression of genes that alter synaptic plasticity in the hypothalamus proceeded in a favorable direction, all because of the repair of the TJ barrier in the mice.^61^ Given that some AD patients endure persistent psychological stress or a state of depression and anxiety, probiotics may be a therapeutic effect in this aspect.

### 4.2 Use of SCFAs for AD

SCFAs include acetate, butyrate, and propionate. Among them, acetate can cross the BBB and decreases the permeability of the BBB.^62^ This may have some ameliorative effect on the possible presence of microbial metabolites that, as previously stated, should not reach the brain due to increased permeability. According to some analyses, *Bifidobacteriun brave strain A1* are associated with acetate and the enhancement of acetate after can mediate the protective effect of *Bifidobacterium breve strain A1*.^59^ Butyrate is a multifunctional molecule that may exert beneficial neuroprotective effects and improve brain health.^63^ In addition, sodium butyrate contributed significantly to improving learning and memory performance in a mouse model of AD, while there was an increase in the expression of learning‐related genes after butyrate treatment.^64^ In normal mice, butyrate also significantly improved memory suggesting that it might be useful for the treatment of AD.^64^ With regard to SCFAs as a whole, they can alter the secretion of hormones in the intestine, such as glucagon‐like peptide‐1, which, in turn, can improve neuroplasticity in the hippocampus.^65^ There is also current literature that suggests that recovery of intestinal SCFAs is likely to help prevent or alleviate the severity of AD symptoms.

### 4.3 Treatment of AD with omega‐3

GM is primarily influenced by Omega‐3 under the following circumstances: (1) Omega‐3 influences the composition of GM; (2) Omega‐3 affects GM metabolic substances, such as proinflammatory mediators, that is, endotoxins (lipopolysaccharides) and IL‐17; (3) Omega‐3 regulates the levels of SCFAs, but its rationale for treating AD is not clear, so we can only assume that it may be used to treat AD based on the following available findings.^66^

A meta‐analysis revealed that DHA levels in the blood are substantially lower in AD patients compared to normal subjects, and colony‐stimulating factor (CSF) concentrations are also lower in AD patients compared to normal subjects.^67^ Some reports also mention that a decrease in brain omega‐3 may be associated with an increase in cognitive aging and AD progression.

Some experiments suggested that omega‐3 can slow down the rate of AD deterioration, that is, postpone the impairment of memory and other functions.^68^ The experiment by Ordóñez‐Gutiérrez et al. investigated the effect of diet on Aβ deposition. It included different fatty acids within each group, with the group with the lowest levels of omega‐3 having the highest Aβ deposits in the brain. Thus, we hypothesize that omega‐3 has an effect on Aβ deposition in the brain. If increasing the amount of omega‐3 in the diet, it may be possible to slow down the increase of Aβ or reduce the amount of Aβ.^69^

### 4.4 Potential use of gut hormones for AD

In the research on type 2 diabetes mellitus (T2DM), a number of researchers have claimed that the development of T2DM due to disturbances in intestinal metabolism is caused by gastrointestinal flora.^70^ They used different intestinal hormones to treat the patients and therefore looked into GM secretion to uncover novel targets for treating AD.^71^, ^72^ As with T2DM, one of the causes of AD is disorders of intestinal metabolism. Through the search for therapeutic approaches to T2DM, we can also consider the use of intestinal hormones as a new approach to AD or find new targets for it.

Increased plasma concentrations of gut hormones, including but not limited to gastric hunger hormone, leptin, gastric inhibitory peptide (GIP), and glucagon‐like peptide‐1 (GLP1), have the potential to improve cognitive function in the brain.^45^ Additionally, gastric hunger levels are found to be negatively correlated with increased proportions of Bacillus/thick‐walled bacteria and Lactobacillus, among others.^73^ Peptide hormones secreted by the intestine have been found to regulate neurological functions, such as learning and memory.^74^ Plasma levels of this hormone were higher in mice treated with the aforementioned mentioned probiotic blend of eight different Gram‐positive bacterial species, VSL#3.^58^ In contrast, GHRELIN has been extensively shown to alleviate memory impairments and prevent synaptic degeneration in animal models of AD, and leptin has been shown to exist as a neurotrophic factor that protects against Aβ oligomer‐induced toxic damage in vitro.^75^, ^76^

## 5 SUMMARY AND PROSPECT

With further exploration of the influence of GM on the neurocentral system via the BGA, there are still great prospects for relying on GM to treat, mitigate, or prevent AD. However, there are still several problems: To begin, there are conflicting findings about whether the increase of gut‐derived LCA in serum and brain tissues is related to AD, and some experiments have shown no detectable increase of LCA in patients’ serum and brain tissues. Second, whether supplementation with omega‐3 FA can improve AD has not yet been determined. Some evidence currently suggests that it is only effective in mild AD, some experiments show that supplementation with omega‐3 FA does not have a significant effect, and some literature even shows that DHA does not work at all.^77^ However, the use of omega‐3 FA for therapeutic purposes is now mostly in animal experiments and less in clinical trials.

The prospects for omega‐3 to become an effective drug for the treatment of AD are great. The currently known effects of omega‐3 include anti‐inflammatory, vasodilatory, and capable of accelerating blood circulation in the brain. Combined with what was said above about many substances entering the brain that should not enter the brain because of altered BBB permeability, it is possible that if blood circulation in the brain is accelerated, it could make it less likely that those substances will enter the brain, thus improving the symptoms of AD or slowing down the onset of AD; in other psychiatric disorders, the phosphorylated structure of cell membranes is altered, and when the level of omega‐3, one of the cell membrane components, is low, it can exacerbate some symptoms, such as aggression in bipolar disorder.^78^ Therefore, increased levels of omega‐3 in the cell membranes of AD patients might improve the symptoms. Also, omega‐3 may provide xiolytic effects, possibly because it regulates anti‐inflammatory factors, which, in turn, increase brain‐derived neurotrophic factor (BDNF), thereby stimulating the synaptic growth of 5‐hydroxytryptaminergic neurons to reduce anxiety or depression.^79^ The current literature reports that a proportion of patients remain in a depressed or low mood during the development of AD in normal individuals, and it has been established that this state of mind influences the progression of AD.^80^, ^81^ In this light, omega‐3 may become a drug to prevent, slow down, and improve the symptoms of AD.

## AUTHOR CONTRIBUTIONS

Xin‐Yan Li analyzed most of the data and wrote the initial draft of the paper. Hao‐Yue Qin contributed to the central idea. Ting‐Ting Li provided corrections and guidance for this article.

## CONFLICT OF INTEREST

The authors declare no conflict of interest.

## ETHICS STATEMENT

The ethics statement is not available.

## TRANSPARENCY STATEMENT

Xin‐Yan Li, Hao‐Yue Qin, and Ting‐Ting Li affirm this manuscript to be original and based on references.

## Data Availability

Data sharing is not applicable to this article as no new data were created or analyzed in this review.
